# Experimental Modelling of the Consequences of Brief Late Gestation Asphyxia on Newborn Lamb Behaviour and Brain Structure

**DOI:** 10.1371/journal.pone.0077377

**Published:** 2013-11-06

**Authors:** Margie Castillo-Melendez, Ana A. Baburamani, Carlos Cabalag, Tamara Yawno, Anissa Witjaksono, Suzie L. Miller, David W. Walker

**Affiliations:** 1 The Ritchie Centre, Monash Institute of Medical Research, Monash University, Clayton, Victoria, Australia; 2 The Department of Obstetrics and Gynaecology, Monash University, Clayton, Victoria, Australia; The Ohio State Unversity, United States of America

## Abstract

Brief but severe asphyxia in late gestation or at the time of birth may lead to neonatal hypoxic ischemic encephalopathy and is associated with long-term neurodevelopmental impairment. We undertook this study to examine the consequences of transient *in utero* asphyxia in late gestation fetal sheep, on the newborn lamb after birth. Surgery was undertaken at 125 days gestation for implantation of fetal catheters and placement of a silastic cuff around the umbilical cord. At 132 days gestation (0.89 term), the cuff was inflated to induce umbilical cord occlusion (UCO), or sham (control). Fetal arterial blood samples were collected for assessment of fetal wellbeing and the pregnancy continued until birth. At birth, behavioral milestones for newborn lambs were recorded over 24 h, after which the lambs were euthanased for brain collection and histopathology assessments. After birth, UCO lambs displayed significant latencies to (i) use all four legs, (ii) attain a standing position, (iii) find the udder, and (iv) successfully suckle - compared to control lambs. Brains of UCO lambs showed widespread pathologies including cell death, white matter disruption, intra-parenchymal hemorrhage and inflammation, which were not observed in full term control brains. UCO resulted in some preterm births, but comparison with age-matched preterm non-UCO control lambs showed that prematurity *per se* was not responsible for the behavioral delays and brain structural abnormalities resulting from the *in utero* asphyxia. These results demonstrate that a single, brief fetal asphyxic episode in late gestation results in significant grey and white matter disruption in the developing brain, and causes significant behavioral delay in newborn lambs. These data are consistent with clinical observations that antenatal asphyxia is causal in the development of neonatal encephalopathy and provide an experimental model to advance our understanding of neuroprotective therapies.

## Introduction

Notwithstanding the recent debate over the appropriateness of the terms Neonatal Encephalopathy and Hypoxic-Ischemic Encephalopathy [1]–[3], there continues to be uncertainty about the contribution of antenatal hypoxic-ischemic events to the functional and structural brain pathology that can arise after birth at term [4], [5]. Clinical and epidemiological studies provide evidence suggesting that a ‘sentinel’ antenatal event may be responsible for the brain injury that appears in some infants after birth, particularly those born preterm, or subsequent to intrauterine growth restriction or maternal infection [6]–[9]. Attempts to understand the etiology of perinatal brain damage and then develop therapeutic strategies to prevent or mitigate these effects is limited by the lack of an appropriate animal model where disturbance of fetal homeostasis *in utero* near term, but before the onset of labor, results in identifiable and quantifiable postnatal neurobehavioral deficits.

We [10], [11], and others [12]–[14] have described the distribution and type of brain injury that arises from brief asphyxia *in utero* in late gestation fetal sheep, produced by transiently occluding the umbilical cord. We have also observed cerebral hypoperfusion and disruption of cortical activity for up to 24 h following umbilical cord occlusion (UCO) [15]. The distribution and type of brain injury that arises from this global fetal asphyxia has been described in detail for the 24–72 h period after the fetal insult [10], [13], [14], but it is not known if the brain pathology partly resolves by the time of birth, or evolves further and then has neurodevelopmental and behavioral consequences for the newborn animal.

The first objective of this study was to determine the effects on lamb survival and behavior of a brief asphyxial insult produced by occluding the umbilical cord at 132 days gestation - considerably before the expected time of parturition (∼147 days). Thus, we chose this gestational age in order to extend our previous work [10], [11], and also to allow sufficient time to pass to determine if functional deficits were present at birth after the asphyxia induced earlier which, if the pregnancy went to term, would be approximately 15 days. We utilised a behavioral testing method for assessing the ability of newborn lambs to resuscitate themselves and reach important behavioral ‘milestones’ after birth, such as gaining posture, locomotion, and success at suckling based on the assessment criteria described by the Scottish Agricultural College and Dwyer and colleagues [16]. The second objective was to document the type and distribution of brain damage present in the newborn brain after the asphyxial event at 132 days gestation. While necrotic and apoptotic death have been described in the fetal sheep brain at 24–72 h following global fetal asphyxia [10], [13], [14], it remains possible that repair processes *in utero* compensate for this damage prior to birth. For example, erythropoietin (EPO) and its receptor [11] and brain derived neurotrophic factor [17] are robustly expressed in the fetal sheep brain following *in utero* asphyxia produced by UCO, and administration of EPO has been suggested as a treatment for perinatal brain damage [18], [19]. Therefore, we examined the postnatal brain 24 h after birth to determine the distribution and type of brain injury that results from a brief asphyxic event approximately 2 weeks before expected term, and to characterize the brain pathology that underlies the neurobehavioral deficits in these neonates.

## Materials and Methods

### Ethics Statement

All experimental procedures had received prior approval from the Monash University School of Biomedical Sciences Animal Ethics Committee, in accordance with the Australian Code of Practice Guidelines for the Care and Use of Animals for Scientific Purposes.

### Animal Care and Surgery

Fourteen pregnant Merino-Border Leicester multiparous 4–6 year-old ewes carrying a single fetus (n = 13 ewes) and one twin pregnancy (full term control) were used in this study. Gestational length was known to within 24 h from the mating records. At 125 days gestation (term is ∼147 days), ewes were given prophylactic antibiotics (500 mg Ibimycin i.v; Douglas Pharmaceuticals; Baulkham Hills, NSW, Australia) and general anesthesia was induced by a bolus i.v injection of 20 ml thiopentone sodium (Pentothal, Rhone Merieux Australia), and anaesthesia was maintained with isoflurane (Isoflo, Abbott Australasia Pty Ltd, Kurnell, Australia) in oxygen/nitrous oxide through an endotracheal tube. Under aseptic conditions, the uterus was identified through a midline incision, and the fetal rump exposed. A polyvinyl catheter (OD - 1.52 mm, ID - 0.86 mm; Critchley Electrical, NSW, Australia) filled with heparin-saline was inserted into one femoral artery and advanced approximately 7.5 cm towards the heart; this catheter was used to obtain arterial blood samples and to record fetal arterial pressure. A second, larger bore catheter was secured onto the fetal skin to measure amniotic fluid pressure. In 7 fetuses an inflatable silastic cuff (type OC16, *In Vivo Metric*, Healdsburg, USA) was secured around the umbilical cord. Immediately before placing the cuff around the umbilical cord, the volume of water needed to fully inflate the cuff and occlude the blood vessels was determined. In the other 7 fetuses the cord was manipulated at surgery but the cuff was not placed around the cord. The fetuses were then replaced in the uterus, the membranes and uterine incision repaired, and the catheters externalized through a small incision in the ewe’s flank. A fetal arterial blood sample (0.5 ml) was collected each day to measure PO_2_, PCO_2_, pH, O_2_ saturation and blood lactate (ABL 700 Series, Radiometer Copenhagen) and after 7 days recovery the experiment was commenced.

### Induction of Fetal Asphyxia by Umbilical Cord Occlusion

At 131 days gestation the fetal femoral and amniotic catheters were connected to solid- state pressure transducers, and fetal arterial pressure was recorded continuously after electronic subtraction of amniotic pressure. Fetal heart rate was determined online from the pulse pressure. All data was recorded continuously on the hard disk of a computer via an analogue-digital converter and Chart software (PowerLab, ADInstruments, Bella Vista, Australia). At 132 days gestation, UCO was undertaken in the 7 fetuses where the umbilical cuff had been implanted, with injection of a volume of sterile water (2–3 ml) into the cuff to inflate it sufficiently to cause complete cessation of blood flow in the umbilical cord. The duration of the occlusion was set at 10 min, but if mean fetal arterial pressure fell to <20 mmHg the cuff was released and the duration of occlusion was recorded. Fetal arterial blood samples (2–3 ml) were taken at −60, −5, 5, 10, 30, 60, 120, 240, 480, and 720 minutes relative to the onset of the cord occlusion at time 0. Fetal heart rate and arterial pressure were recorded before, during and for 24 h after the cord occlusion, after which the ewe was moved to a large ‘lambing pen’ until the onset of labor. Where no cuff had been implanted, the ewes were also moved to the lambing pen on day 133 of gestation. Fetal blood samples (1–1.5 ml) were taken each day to monitor the condition of the fetuses.

### Induction of Preterm Birth

Because occlusion of the umbilical cord resulted in early delivery in 5 of the 7 UCO lambs compared to the sham-occluded group (see Results), a further group of 3 (one singleton and two twin) ewes was introduced to the study where labor was induced by intramuscular injection of betamethasone (5.7 mg, Celestone Chronodose; Schering Plough, Australia) at 12∶00 h, followed by Epostane (50 mg in 2 ml ethanol) at 17∶00 h on day 136 of gestation. This led to the onset of premature labor 36–48 h following the Epostane injection [20], [21]. If labor had not commenced after 48 h (i.e., by 138 days gestation), a further injection of Epostane (50 mg) was given at 17∶00 h on that day.

### Birth of the Lamb and Scoring of Lamb Behavior

Four closed-circuit cameras (SW224-MDN, Swann Security Products) with infrared night vision were mounted on the ceiling directly above each lambing pen and were used to continuously monitor the ewe for signs of labor, birth and lamb behavior. At the onset of the final stages of labor when membranes were visible at the vulva and/or there was loss of amniotic fluid, at least two investigators were present to assist the ewe if necessary, to cut and secure the catheters when the lamb was delivered and to remove the umbilical cuff immediately after the lamb was clear of the birth canal.

We utilised a neurobehavioral assessment based on observations previously conducted in sheep to monitor their wellbeing following birth [16]. The time taken to reach normal lamb behavioral milestones immediately after birth (head lift and shake; first use of hind limbs; first use of four legs; standing; finding the udder, suckling) were assessed with time ‘0’ being the time at which all parts of the lamb had completely cleared the birth canal. If a lamb did not achieve the final milestone of suckling within the first 4 h after birth, it was bottle-fed, as it was expected that lambs should have performed these important behaviors within this time frame [16], [22], [23]. Once lambs had achieved the final assessment milestone, or at 4 h post delivery if milestones had not been met, the lamb was weighed and immediately returned to its mother. The amount of the time between 4 and 23 h that the lamb was standing and/or walking (denoted as ‘active’), and the time spent at rest (sitting, apparently sleep) were determined in relation to the total amount of this recorded time (i.e., 19 h). Lambs that were not able to self-feed from their mother by 4 h after birth were bottle-fed at 4 h intervals until 24 h of age when, at this time, all lambs were euthanized by intravenous injections of pentobarbitone sodium (Lethabarb, Virbac Pty. Ltd., Peakhurst, NSW, Australia).

### Histology and Immunohistochemistry

The brain was removed and cut sagittally into left and right hemispheres and each half was cut coronally into approx. 5 mm thick blocks. (The cerebellum was removed, and together with the pons and medulla were not examined in this study). All blocks were immersion-fixed in 4% paraformaldehyde for 48 h, and then immersed in 70% ethanol for 3 to 4 h, cleared in sulphur-free xylene and paraffin embedded. Sections of forebrain were cut at 10 µm thickness and stained for morphological changes with hematoxylin and eosin (H&E). Luxol Fast Blue was used to assess myelin organization and Mallory trichrome staining was used to assess the presence of brain hemorrhage and micro-bleeds, using criteria published elsewhere [24]. Immunohistochemistry was then carried out using antibodies raised against glial fibrillary acidic protein (GFAP; 1∶400; Sigma-Aldrich, USA) for identification of astrogliosis; peroxidase-labeled lectin (1∶200; Sigma-Aldrich, USA) to identify microglia, macrophages, and inflammatory cell infiltration; albumin (1∶1000; Accurate Chemical & Scientific Corporation, USA) to detect areas of blood-brain-barrier (BBB) disruption; cleaved caspase-3 (1∶1000; R & D Systems, Minneapolis, MN, USA) to identify apoptosis; and CNPase (1∶200; Sigma-Aldrich, USA) to identify immature and mature oligodendrocytes and the density of myelin sheaths.

### Qualitative Analysis of Brain Injury

Examination and scoring of neuropathology outcomes were carried out by an investigator blinded to the treatment (MCM). The brain regions of interest included the frontal cortex, parasagittal cortex and lateral ventricle, subcortical and periventricular white matter, hippocampus and basal ganglia/thalamus. Photomicrographs were taken under light microscopy (Olympus, Tokyo, Japan) at 400x magnification and examined under light microscopy. Neuropathologies were noted, scored and/or counted using a 3 point scoring system based on previously published criteria [25], with the further introduction of a mild and severe group for the pathology. The scoring was follows: score 0 = absent, 1 = present mild pathology, or 2 = present severe pathology. This scoring system was applied to determine: (i) presence of necrotic cells (eosinophilic cytoplasm or shrunken pyknotic nuclei); (ii) lesions that were either cystic or diffuse, and parenchymal abnormalities with areas of pallor or the presence of vacuoles; (iii) disordered or reduced myelination; iv) presence of inflammatory cells and astrocytic activation; and (v) vascular integrity comprising scoring for intra-parenchymal bleeds, albumin extravasation and/or perivascular astrocyte abnormalities. For each animal, an average score for each neuropathology was derived across all regions examined. In addition, cell counting of single-label immunopositive cleaved caspase-3 positive cells (for detection of activated caspase-3, a principal final step in the apoptotic pathway) was determined in parasagittal cortex, corpus callosum, external capsule, subventricular zone and periventricular white matter under light microscopy (Olympus, Tokyo, Japan) at 400x magnification. CNPase-positive cell counts of immature/mature myelinating oligodendrocytes and the density of CNPase-immunoreactive myelinated fibres were measured in corpus callosum, external capsule, internal capsule, subcortical white matter and periventricular white matter. The densitometric calculation was performed using a computerized image-analysis system (ImageJ version 10.2, NIH, Bethesda, Maryland, USA) that read optical density as grey levels, at 400x magnification. An investigator (TY) blinded to the experimental groups selected the threshold levels of detection, where non-specific background was easily identifiable and the threshold level remained constant for all images within a given histochemical run. For cell counts and scoring, two sections of each brain region per animal were examined, and the number of immunopositive cells per region, or neuropathology score, was calculated using an average of three fields of view per area, and the results were averaged across the animals in each group.

### Statistical Analyses

A Shapiro-Wilks test for normality and Levene’s test for homogeneity of variance were run on all data. Physiological parameters (fetal blood gases, blood lactate, blood pressure and heart rate) were analyzed by SPSS software using a repeated measures one-way ANOVA, with a ‘Contrast (simple)’ post hoc test to compare to pre-UCO values. Body and organ weights were analyzed using a one-way ANOVA, and a Tukey’s post hoc test. Behavioral data for lambs in the three study groups (UCO lambs, full term control lambs, preterm control lambs) were analyzed using a Kruskal Wallis one-way ANOVA and if significant differences were present, a post hoc analysis using a Mann-Whitney U test was done. Caspase-3 and CNPase data were analysed using a one-way ANOVA and if significant, a student newman keul post hoc test was run. Neuropathological scores were analysed using Kruskal-Wallis test followed by a Dunn’s multiple comparisons post hoc test. All statistical analyses were conducted using SPSS (v18 for Windows). Values are shown as mean ± standard error (SEM). Significance was set at *p*<0.05.

## Results

### Fetal Asphyxia

UCO for 10 mins at 132 days of gestation caused significant hypoxemia, hypercapnia, and acidosis (Table 1). However, by 2 h post-UCO the fetal arterial PO_2_, O_2_ saturation, PCO_2_, and pH had recovered and were not different from the pre-occlusion values. The increase in plasma lactate concentrations returned to normal values more slowly; by 4 h after UCO the base excess was not different from the control value, and the fetal arterial blood lactate concentration remained slightly but significantly higher than the pre-occlusion value until 24 h after the UCO (Table 1). Fetal arterial pressure and heart rate were increased as a result of the UCO, but from 4 h after the UCO arterial pressure was normal, and heart rate was not different from the pre-UCO values from 2 h after UCO. Haematocrit was increased at the end of the UCO, and slightly decreased at 2, 4 and 8 h after UCO, but was not different from the pre-UCO value of 32.16±0.87% at 12, 24, or 48 h after UCO (Table 1).

**Table 1:** Fetal arterial blood gases, pH, hematocrit, blood lactate, and arterial pressure and heart rate before (−1 h), at the end of (+10 mins), and 1 to 48 h after occlusion of the umbilical cord at 132 days gestation.

Time relative toonset of UCO	−1 h	+10 mins	+1 h	+2 h	+4 h	+8 h	+12 h	+24 h (n = 4)	+48 h (n = 4)
***p*** **O_2_ (mmHg)**	20.2±0.7	4.9±1.6*	21.2±0.6	20.4±0.5	18.6±1.5	19.3±1.0	18.2±1.1	19.4±1.3	21.0±1.0
***p*** **CO_2_ (mmHg)**	55.4±1.0	126.2±14.1*	53.5±0.9	51.4±1.0*	52.2±1.2*	54.1±1.1	53.8±0.6	54.0±1.2	53.3±1.4
**O_2_ saturation (%)**	48.5±3.2	5.0±2.4*	47.6±2.7	48.3±2.3	44.1±5.5	44.1±4.8	41.2±4.5	46.1±4.4	48.9±4.0
**pH**	7.35±0.01	6.95±0.07*	7.28±0.02*	7.33±0.01	7.35±0.01	7.35±0.01	7.35±0.01	7.35±0.00	7.34±0.01
**Blood lactate (mmol/L)**	1.6±0.2	8.9±1.6*	6.6±1.2*	4.6±0.8*	3.5±0.7*	3.3±0.8	2.8±0.7	1.7±0.2	1.7±0.3
**Base Excess (mmol/L)**	3.3±0.4	−10.0±2.5*	−2.7±1.0*	0.1±0.7*	1.9±0.4*	3.0±0.4	3.0±0.4	2.8±0.4	2.3±0.6
**Hematocrit**	32.2±0.9	34.7±0.8*	31.5±1.3	30.7±1.1*	30.6±1.2*	30.3±1.0*	30.5±1.0	31.0±0.6	31.5±0.3
**Arterial pressure (mmHg)**	43.1±2.6	47.6±1.8	48.1±2.9	47.0±2.7*	41.7±0.9	41.2±0.7	42.1±0.7	n/a	n/a
**Heart rate (beats/min)**	157.1±3.1	212.7±39.6	185.0±7.7*	157.0±5.9	155.9±3.1	154.2±3.8	157.0±4.5	n/a	n/a

Data shown as mean ± SEM, n = 5 throughout, except for +24 h and +48 h (n = 4).

* *p*<0.05, compared to values at −1 h. (n/a = data not available).

### Pregnancy Outcome

Control lambs were born between 147 and 151 days gestation (147.5±0.9 days), whereas UCO lambs delivered between 134 and 147 days (139.7±2.2 days; *p*<0.05; Table 2). One lamb in the UCO group was born preterm at 134 days gestation, showed poor respiration and was euthanized at 1 h after delivery; all remaining lambs (n = 6) were euthanized 24 h after birth and are included in behavior and neuropathology analyses. The birth weight of UCO lambs was 3.51±0.32 kg, significantly less than the weight of lambs in the full-term control group (4.87±0.34 kg; *p*<0.05). After injection of Epostane to induce labor, preterm control lambs were all born at 138–139 days, with a mean birth weight of 3.74±0.13 kg (*p* = 0.6 vs UCO lambs and *p*<0.05 vs full-term control lambs). Organ weights relative to body weight were not different between the 3 groups, except adrenal gland relative to body weight was significantly increased in UCO lambs compared to control full-term and preterm control lambs (Table 2). There were no overt signs of infection or sepsis at the birth of any lamb, and meconium staining was not more intense than usually observed in fetal sheep at term.

**Table 2:** Gestational age, and body weights at birth for lambs born after a normal pregnancy (Full-term control, n = 8), after experiencing 10 mins of cord occlusion *in utero* at 132 days gestation (UCO, n = 6), or after induction of preterm birth by treatment of the ewe with betamethasone and Epostane at 136 days gestation (Preterm control, n = 5).

	Full Term Control (n = 8)	Preterm Control (n = 5)	UCO (n = 6)
**Median (range) gestational age at delivery (days)**	148 (144–151)	138 (138–139)	137.5 (134–147)
**Birth weight (kg)**	4.87±0.34	3.74±0.16	3.51±0.32
**Male/female Ratio**	2/6	2/3	2/4
**Nose-rump length (cm)**	66.1 (n = 2)	61.1±1.73	59±2.21 (n = 4)
**Organ weights (g)**			
** Brain**	54.43±1.15	47.61±1.08*	46.81±1.73*
** Heart**	39.67±2.23	41.52±5.74	30.18±6.88
** Liver**	153.31±6.31 (n = 4)	93.61±10.04*	93.61±15.02
** Brown Fat**	27.21±5.21	12.99±2.54	19.42±4.42
** Kidney (left and right)**	32.70±3.22	24.01±1.11	24.72±3.62
** Adrenal Glands (left & right)**	0.876±0.071	0.697±0.08	0.846±0.090
**Organ (g) - body weight (kg) ratio**			
** Brain**	11.55±0.86	12.82±0.63	13.72±0.95
** Heart**	8.21±0.23	10.95±1.08	8.94±2.07
** Liver**	27.85±0.95	24.75±1.68	27.98±6.28
** Brown Fat**	5.25±0.76	3.57±0.80	5.31±0.94
** Kidney (left and right)**	6.80±0.69	6.39±0.06	6.90±0.52
** Adrenal Glands (left & right)**	0.181±0.0133	0.185±0.0144	0.246±0.0236*

Age at delivery shown as median (range), all other data shown as mean ± SEM.

* indicates *p*<0.05, compared to full-term controls.

† indicates P<0.05 UCO compared to preterm group. Abbreviation: UCO, umbilical cord occlusion.

### Postnatal Behavior

**Figure 1:**
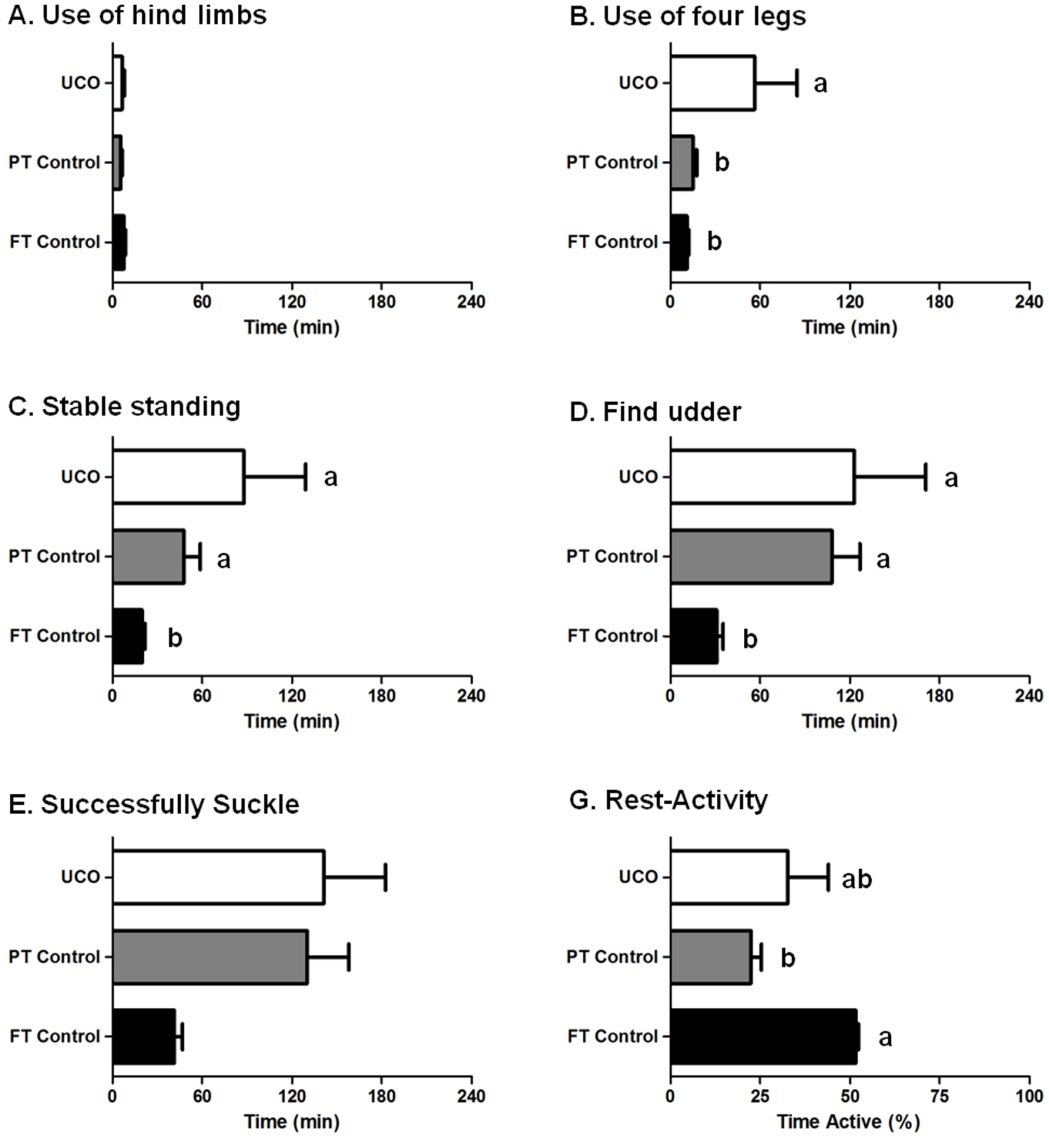
Postnatal lamb behavior was observed from the time that lambs fully cleared the birth canal (time 0). Time taken to use hind limbs (A), four legs (B), stand stable for >5 sec (C), find the udder (D) and successfully suckle (E) for full-term control (n = 6), preterm control (n = 5) and UCO lambs (n = 5) were recorded. The percentage of time spent active from 4 to 23 h was also monitored (G). Data are expressed as mean ± SEM. *p*<0.05.

A closed-circuit video record was obtained in 6 of 8 full-term control lambs, 5 of 6 UCO lambs, and 5 of 5 preterm control lambs. The times taken for lambs to attain behavioral milestones after birth are shown in Figure 1. With birth as time 0, full-term control lambs first attempted to use their hindlimbs at 7.7±1.0 min (Fig. 1A), and on average had achieved a stable standing position on 4 legs for >5 seconds at 19.6±2.3 min after birth (Fig. 1C). After standing, control lambs immediately began looking for the udder. They successfully found the udder at 30.9±4.7 min (Fig. 1D) and were attached to the teat and suckling at 41.1±6.2 min after birth (Fig. 1E). Compared to full-term control lambs, preterm control lambs were significantly delayed in the behavioural milestones of achieving a stable standing position (47.7±10.1 min, *p* = 0.045; Fig. 1C) and finding the udder (108.0±17.2 min, p = 0.011; Fig. 1D). As observed in preterm control lambs, UCO lambs were also significantly delayed in achieving a stable standing position (87.8±41.3 min, p = 0.018; Fig. 1C), in which one lamb failed to achieve this in 4 h and was thereby given a time of 4 h, and finding the udder (123.0±47.8 min, p = 0.017; Fig. 1D) compared to full-term control lambs, however they also demonstrated significant latency compared to both full-term (p = 0.006) and preterm (p = 0.028) control groups in their first attempt to use 4 legs to stand (Fig. 1B). Both preterm control lambs and UCO lambs tended towards latencies in the milestone of attaching to the teat and suckling (130.3±25.4; p = 0.01, and 141.1±41.3; p = 0.03, respectively vs full-term control), however it should also be noted that two lambs in the UCO group had not suckled at 4 h, and were then bottle-fed; these lambs were assigned a ‘time to suckle’ of 4 h. The percentage of time spent active was monitored in full-term (n = 5), preterm (n = 3) and UCO lambs (n = 4). Preterm control lambs spent significantly less time active (22.5±2.9%) compared to full term controls (55.7±0.7%) and UCO lambs (32.7±11.4%; Fig. 1G). Although the EEG was not measured in this study, there were no outward signs of seizures in any of the UCO or control lambs.

### Immunohistochemistry & Histopathology – Brain Injury

#### i) Gross pathology

**Figure 2:**
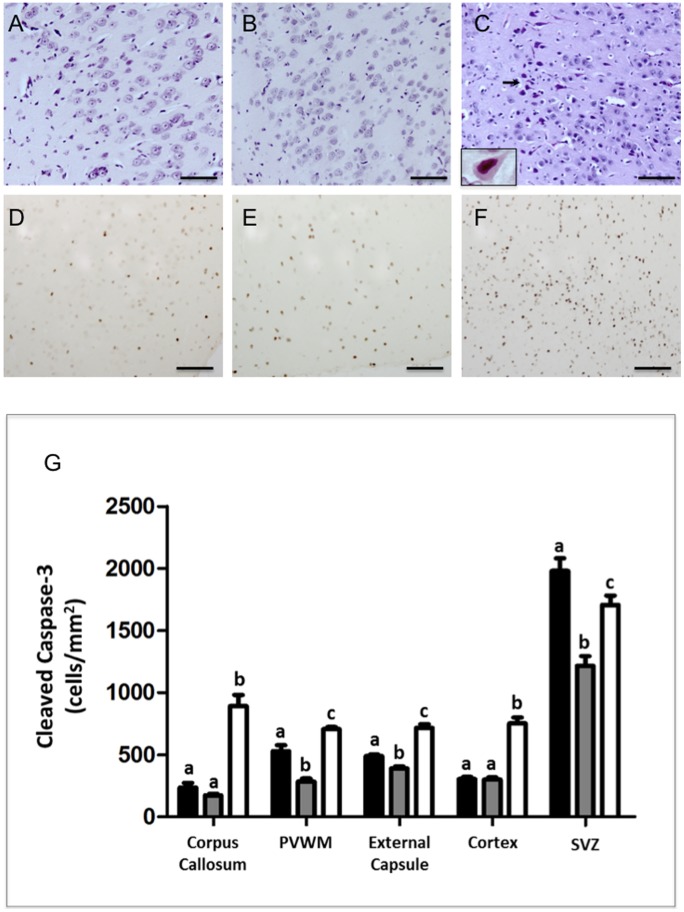
Hematoxylin & Eosin (A–C). Few degenerating cells were seen in the cortical gray matter of full-term (A) and preterm (B) control lambs. UCO lambs showed extensive neuronal injury displaying feature of apoptosis seen by H&E staining as scattered dark, shrunken cells with pyknotic or small, densely staining nuclei and eosinophilic cytoplasm (black arrows C). The insert in panel C is a high power micrograph showing a neuron exhibiting ischemic morphology (eosinophilia, and nuclear pyknosis). Panels D–F show activated Caspase-3 immunoreativity in the cortex of a full term (D), preterm (E) and UCO (F) lambs. Note the increased immunoreactivity of activated Caspase-3 in the cortex of UCO lambs compared with both control groups. Scale bars –50 µm. Panel G: Quantitative results show cleaved caspase-3 cell counts in the corpus callosum, periventricular white matter (PVWM), external capsule, cortex and subventricular zone (SVZ) for full-term control (n = 5), preterm control (n = 5) and UCO lambs (n = 6) (G). Data are expressed as mean ± SEM. *p*<0.05. White, gray and black bars represent full term, pre term and UCO lambs respectively.

**Cell death:** Within brains from UCO animals, there were large numbers of cells demonstrating typical pyknotic features of shrunken, eosinophilic cytoplasm, pyknotic nuclei and chromatin condensation (Fig. 2C), assessed with H&E staining. Degenerative cells were observed with pale swollen cytoplasm, vacuolated nuclei, and apparent compression of the Nissl substance to the rim of the cytoplasm of the cell were also evident (Fig. 2C, insert). Necrotic/pyknotic cells were rarely observed in full-term or preterm control brains, but following UCO necrotic/pyknotic cells were widely distributed in cortical grey (Fig. 2C) and white matter, periventricular white matter, subventricular zone, corpus callosum, striatum (caudate nucleus and putamen), thalamus, external capsule, and hippocampal CA1 and CA3, giving a combined average neuropathology score of 2.0±0.01 for the UCO group compared to zero for both the full-term and preterm control groups (*p*<0.01; Table 3). Cellular apoptosis was quantified by counting cleaved caspase-3 immunopositive cells, and it was observed that preterm control brains demonstrated a significant reduction in the number of caspase-3 stained cells within the external capsule, periventricular white matter and subventricular zone, compared to full-term control brains (Fig. 2G; p<0.05). Following UCO, there was a significant increase in numbers of caspase-3-positive cells across all areas examined compared to full-term and preterm control brains, with the exception of the subventricular zone, where there was a significant reduction in apoptotic caspase-3-positive cell counts compared with full-term control (Fig. 2G).

**Table 3:** Histopathological features seen 24 hours after birth in the brain of lambs born at full term, preterm, or after UCO at 132 days gestation.

*Gross Pathology*	Full TermControl (n = 6)	PretermControl (n = 5)	UCO (n = 6)
***Cell Death/damage***			
Apoptosis	0.67±0.21	1.00±0.08	2.00±0.01** ^,^ §§
Necrosis	0	0	2.00±0.01** ^,^ §§
Cystic Lesions/Parenchymal Injury	0	0	1.90±0.1** ^,^ §§
***White Matter Abnormalities/Myelin disruption***			
Luxol Fast Blue	0.17±0.02	0.20±0.10	2.00±0.00** ^,^ §§
***Inflammation***			
Inflammatory Cells Infiltrate	0.50±0.20	0.57±0.19	2.00±0.02** ^,^ §§
Reactive astrocytes	0	0.40±0.20	1.70±0.2** ^,^ §
***Vascular Integrity***			
Albumin Extravasation	0	0.40±0.24	2.00±0.00** ^,^ §§
Microbleeds	0.70±0.20	0.60±0.24	1.83±0.17** ^,^ §
Loss of Vascular Astrocytic Processes	0.14±0.14	0.40±0.24	1.83±0.17** ^,^ §

Neuropathology features were divided into 4 categories: **(1)** Cell death/damage **(2)** White matter abnormalities **(3)** Inflammation and **(4)** Vascular Integrity. Brain sections were examined using light microscopy at 400x or 1000x magnification following staining with Hematoxylin-Eosin or Luxol Fast Blue. Immunohistochemical staining was with albumin (blood brain barrier permeability) or GFAP (astrocytic marker). Specific scores were given to pathologies seen in the cortical gray and white matter, periventricular white matter, striatum, thalamus and cerebellum and an average score was given to each brain for each pathology. An overall score was then given to each experimental group based on the observed pathologies. Bleeding in the brain 24 hours following birth detected by Mallory’s Trichrome staining. Scores from 0 to 3 were given to each of the pathologies depending on the severity of the pathology. 0 = absent; 1 = mild; 2 = severe. Data are shown as mean ± SEM. Abbreviation: UCO, umbilical cord occlusion.

* p<0.05;

** p<0.01;

*** p<0.001 UCO lambs vs full term controls.

§ p<0.05;

§§ p<0.01 preterm controls vs UCO lambs.

#### Cystic lesions

**Figure 3:**
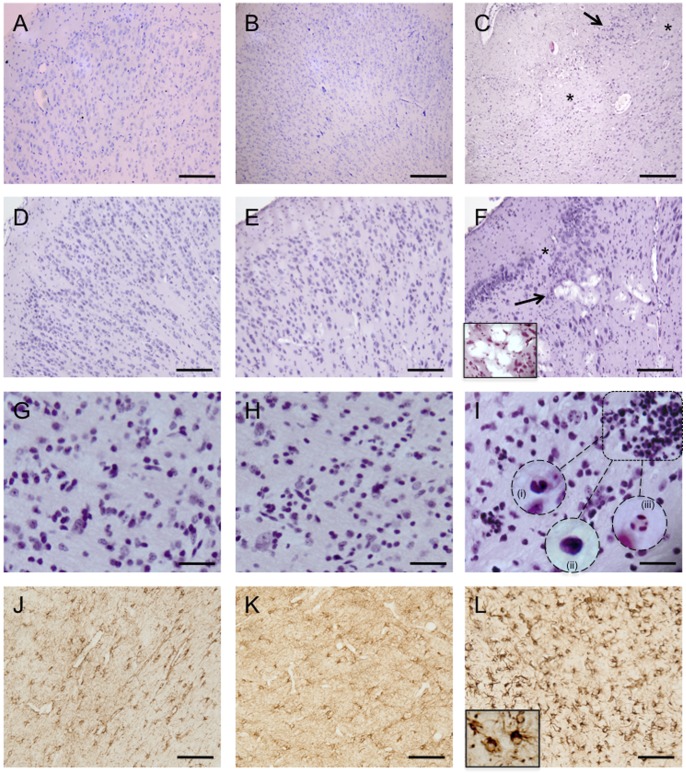
Hematoxylin & Eosin staining of full term control (A, D & G), preterm control (B, E & H), and UCO lambs (C, F &I). Normal cytoarchitecture of the cerebral cortex was seen in both full-term (A) and preterm brains (B). In UCO brains (C) subtle alterations in the cellular composition and spatial arrangement of neurons was seen throughout the cortical gray matter. Extensive neuronal injury (arrows) in the cortical gray matter of UCO lambs as well as areas devoid of neurons (asterisks). D & E show normal pathology in the cortex of full term and preterm control lambs respectively. In UCO lambs (F) some other cellular degenerative changes were observed, such as vacuolation of brain parenchyma (arrow). The insert in panel F shows a high power view of the vacuolar degeneration, histologic features consistent with hypoxic/ischemic changes. Inflammatory cell infiltration was seen in the periventricular white matter of UCO lambs (I), as marked by the box, which showed the morphological appearance of eosinophils (i), lymphocytes (ii) and neutrophils (iii) (inserts in I). These were not seen in full term (G) or preterm control lambs (H). Panels J, K & L are representative images of glial fibrillary acidic protein (GFAP) staining in the periventricular white matter of full term control, preterm control and UCO lambs respectively. UCO lambs displayed reactive astrocytosis. Note the dense staining of the enlarged cell bodies and the highlighted cell processes shown in the high power insert in panel L. Scale bars: A–F = 100 µm, G–L = 50 µm.

Within 3 of 6 UCO brains, H&E staining demonstrated the presence of large, a cellular cystic areas in cortical grey and white matter (Fig. 3, panels C & F), and microscopic lesions where tissue degeneration, loss of cytoarchitecture and tissue homogeneity (Fig. 3, panels C & F), and cavitation (vacuolization) of the tissue was observed (see Fig. 3F). Cystic lesions were not observed in full-term or preterm control brains and tissue degeneration was rarely observed (scores 0.1±0.01 and 0, respectively), compared to a neuropathology score of 1.9±0.1 in UCO (p<0.01; Table 3).

#### ii) White matter

**Figure 4:**
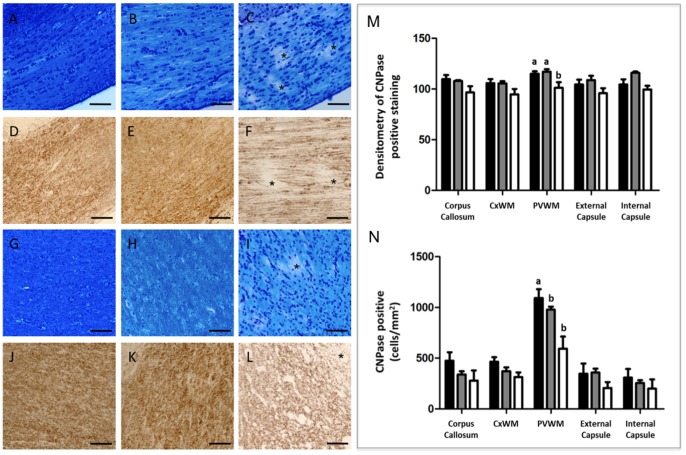
Luxol fast blue staining on brain sections of full term control (A), preterm control (B) and UCO lamb (C) in the corpus callosum. Myelin irregularities (disruption) seen as patchiness (asterisks in C) were detected only in UCO lambs. Panels D–F show CNPase immunohistochemistry in the corpus callosum of a full term (D), preterm (E) and UCO lamb, confirmed myelin disruption seen as patchy staining (asterisks). Myelin disruption was also seen in the periventicular white matter of UCO lambs both with luxol fast blue (I) and CNPase (L) (asterisks). Myelination in full term (G & J) and preterm (H & K) appeared to be intact with both stains. Scale bars = 50 µm. Quantitative results show densitometry analysis of CNPase stained myelination (M) and CNPase positive cell bodies (N) in the corpus callosum, subcortical (CxWM) and periventricular white matter (PVWM), external and internal capsule for full-term control (n = 8), preterm control (n = 4) and UCO lambs (n = 5). Data are expressed as mean ± SEM. *p*<0.05. White, gray and black bars represent full term, pre term and UCO lambs respectively.

Luxol Fast blue histochemistry demonstrated significant loss of myelin in the corpus callosum, subcortical and periventricular white matter of all UCO brains (Fig. 4 panels C & I); neuropathology score 2.0±0.0) compared with full-term (0.17±0.02) and preterm control lambs (0.2±0.1; Table 3; Fig. 4A, B, G & H; p<0.001). Loss of myelin was confirmed and was quantified using CNPase immunohistochemistry, wherein the density of CNPase-positive myelin fibers was decreased within the periventricular white matter by 12% (Fig. 4M, *p* = 0.016) in UCO brains, compared to both full-term and preterm controls (Fig. 4F & L). Within the periventricular white matter of UCO animals there was also a 45% decrease in the counts of immature/mature CNPase-positive oligodendrocytes (Fig. 4N, p = 0.005), compared to full-term controls. There was no difference in the number of CNPase-positive oligodendrocyte cell bodies across the 3 experimental groups, in any other brain region (Fig. 4N).

#### iii) Brain inflammation

The presence of inflammatory infiltrate was noted in UCO brains, particularly in the subventricular zone, corpus callosum, and periventricular and cortical white matter (Fig. 3I). The infiltrates were found in close association with the cystic lesions described above. These cells showed the morphological features of esonophils, lymphocytes and neutrophils (Fig. 3I inserts) as well as macrophages. In full term and preterm control brains, only a small number of cells showing macrophage morphology were observed. Neuropathology scoring determined that inflammatory cell infiltration was significantly increased in UCO brains (2.0±0.2) compared to full-term (0.5±0.2) and preterm control brains (0.57±0.19; *p*<0.01; Table 3). Although some macrophage infiltration was seen in both the full-term and preterm control brains, these cells were scattered and were not clustered. Similarly, reactive astrocytes (determined by GFAP immunohistochemistry) were only seen in the brains of UCO lambs (Fig. 3L; Table 3).

#### iv) Vascular integrity

**Figure 5:**
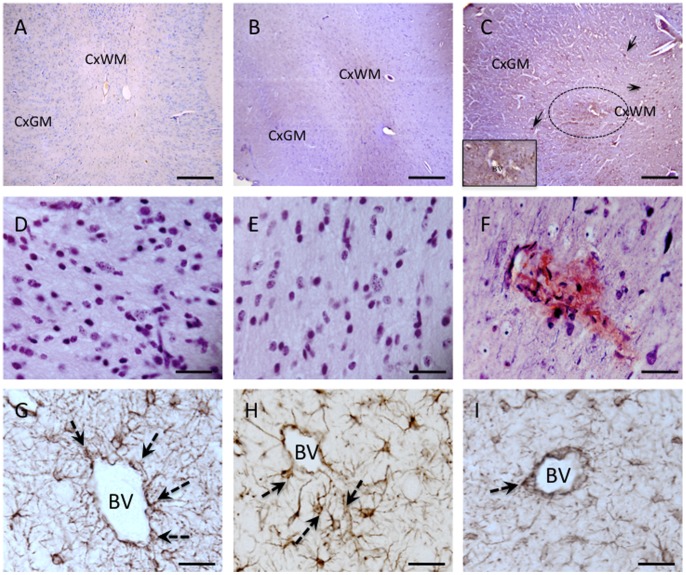
Photomicrograph showing changes to the cerebrovasculature following UCO. Panels A–C show albumin staining in the cortical gray (CxGM) and subcortical white matter (CxWM) of a full term (A), a preterm (B) and a UCO lambs (C). Albumin extravasation (brown staining) consistent with blood brain barrier permeability disruption was observed throughout the brain in UCO lambs (C). Note the positive albumin staining around a blood vessels (circle), as well as in cells (arrows). The insert in C is a high power photomicrograph showing the albumin staining surrounding a blood vessel (BV), as well as albumin positive cells, We observed moderate levels of albumin extravasation in some pre tem control lambs (B), while no albumin staining was noted in brains of full term control lambs. Panels D, E and F show Mallory trichrome staining in the periventricular white matter of a full term (D), preterm (E) and UCO lamb (F). Microbleeds were seen in UCO brains shown by degradation products of hemorrhage staining a muddy brown. No microbleeds were detected in full term or preterm control brains. G–I show GFAP immunohistochemistry and show normal perivascular astrocytes in the periventricular white matter of a full term (G) and preterm lamb (H); while blood vessels in UCO lamb were often seen to be devoid of astrocytic contact (I). BV = blood vessel. Scale bars: A–C = 100 µm; D–I = 20 µm.

A striking finding was the presence of small bleeds within basal ganglia in all 6 UCO lamb brains and periventricular whfite matter in 3 of 6 UCO brain (overall neuropathology score 1.83±0.17; Fig. 5F) compared to the incidence in brains from full-term (0.7±0.2; P<0.05) and preterm control fetuses (0.6±0.24; P<0.05; Table 3). We utilised albumin staining to examine plasma protein extravasation into the brain parenchyma, indicative of BBB leak. In the full-term control lambs, there was no positive albumin staining within the brain (score = 0; Table 3), and 1 of 5 preterm control lambs demonstrated albumin presence within endothelium and smooth muscle of blood vessels (0.4±0.2; Table 3; Fig. 5B). In contrast, all 6 of 6 UCO lamb brains showed widespread and strong albumin staining, particularly in the cortical and periventricular white matter (Table 3, Fig. 5C), cortical grey matter, subventricular zone and basal ganglia (neuropathology score 2.0±0.0; p<0.01). Albumin staining in UCO lamb brains was often associated with loss of perivascular GFAP-positive astrocytic processes (Fig. 5I). The neuropathology score for astrocyte pathology, including reactive astrocytosis together with of perivascular processes, was significantly increased following UCO (1.83±0.17) compared to full-term and preterm control brains (0.4±0.4 and 0.4±0.24, respectively; *p*<0.01; Table 3).

## Discussion

In this study we show that a brief fetal asphyxia *in utero* late in gestation increases the probability of preterm birth, and the lambs have significant behavioral deficits following birth that appear to arise from the underlying neuropathology caused by the asphyxia, and not from the preterm birth *per se*. Immunohistochemical and histological analysis confirmed previous findings in fetal sheep that certain brain areas are vulnerable to hypoxic damage in late gestation; viz., the corpus callosum, subcortical white matter, striatal regions responsible for sensorimotor integration and movement control [13], [26]–[28], consistent with the view that acute antepartum hypoxic events can induce brain damage that manifest as neuromotor and cognitive disabilities in the newborn.

Lambs are precocious and their ‘self-resuscitation’ at birth can be characterised as accomplishing a stepwise series of milestones [29], [30] which, as seen in full-term control lambs, are all achieved by 1 h after birth. Lambs born following UCO showed significant delays in achieving these early milestones, with significant latency to their first use of all four legs to stand, achieving a stable standing position, and first finding of the udder, compared to the control lambs. Preterm lambs also demonstrated some delay in achieving behavioural milestones after birth (e.g., achieving a stable standing position, finding the udder) in which they were delayed compared to full-term controls and were not different to UCO lambs. These findings in the preterm lambs are consistent with human studies of late preterm babies who also show neurodevelopmental delays, which may be due to structural immaturity of the brain [31]. The preterm lambs were also less active overall compared to the term control lambs, which might reflect reduced milk intake as a result of the delayed success at finding the udder and poor sucking.

The UCO lambs were also significantly delayed in their success at using all four legs to stand, compared to both the full-term control group and the preterm control group. This delay in achieving a more complex motor function has also been seen in neonatal primates subjected to fetal hypoxia near term [32]. Similarly, a rabbit model of cerebral palsy using fetal hypoxia/ischemia arising from occluding uterine artery blood flow has reported an inability of the newborn kits to feed, necessitating the use of a oro-gastric catheter [33]. Early sucking is imperative to allow for the ingestion of colostrum as quickly as possible after birth to provide passive immunity in the form of immunoglobulins that pass into the blood stream from the gut [34]. At a later time, depletion of brown fat and glycogen stores in the liver, and skeletal and cardiac muscle require that oral feeding is established to ensure that the newborn has a means to maintain plasma glucose and amino acid concentrations in their normal range. Thus, the delays in achieving these normal behavioral milestones would likely have profound effects on both the short- and long-term wellbeing of these lambs.

The asphyxia insult produced by UCO at 132 days gestation resulted in a number of neuropathological changes, including neuronal necrosis, increased apoptotic cell counts, and diffuse grey and white matter lesions with inflammatory cell infiltrates that included various types of inflammatory cells including neutrophils, lymphocytes, and macrophages. Although some macrophage infiltration and microglial aggregation was seen in the brains of the full-term and preterm control lambs, these cells were scattered and always of low incidence. In contrast, dense arrays of leukocyte infiltration (neutrophils, esoniphils, lymphocytes) were found in the brain only in UCO lambs 24 h after birth, consistent with CNS inflammation. This is of particular importance as neutrophils are typically the first to infiltrate the brain following hypoxia-ischemia, followed by macrophages and lymphocytes [35], [36]. The damaging role of infiltrating neutrophils in ischemia has been demonstrated by studies where either neutrophil depletion [36] or administration of anti-adhesion molecules [37] reversed the localized hypoperfusion, BBB disruption, and the release of free radicals, proteinases, and other cytotoxins seen after adult stroke. GFAP immunohistochemistry showed increased astrocyte reactivity in some regions, and loss of contact of astrocytic processes with blood vessels, suggesting disruption of the BBB. This was confirmed with examination of albumin extravasation in UCO lambs, which was increased compared to both control groups. Disruption of the BBB is an early and prominent feature of CNS inflammation, and in the developing brain following hypoxia it has been shown to lead to structural alterations involving dendrites and axons [38]. Both the cerebrovascular endothelium and adjacent astrocytic end-feet form the functional BBB via production of factors that promote or restrict the passage of substances between blood and the parynchyma of the brain [39], [40]. Many of the morphological astrocyte changes we observed (astrogliosis, loss of perivascular astrocytic processes) are also consistent with cerebral inflammation and increased BBB permeability. Using a micro-array based approach, it has been elegantly shown that inflammation leads to altered astrocyte gene expression to favour a state of increased vascular permeability, while also down-regulating the expression of anti-permeability factors [41].

Parenchymal abnormalities such as vacuolization were also observed in the post-UCO brains, but not in either the full-term or preterm control lambs. These abnormalities were relatively widespread in cortical gray and white matter. This is consistent with cranial ultrasound and MRI findings in human infants showing parenchymal injuries and lesions to these brain regions at term [42], and with injuries seen in experimental animals [43].

A striking histopathological finding was the presence of bleeds in the brains of UCO lambs, particularly in the thalamus. Although micro-bleeds were seen both in preterm and full-term control brains – thus suggesting that birth itself might induce a certain amount of intra-parenchymal haemorrhage – these were only observed around small blood vessels, and serious bleeds with parenchymal involvement were seen only in the brains of lambs from the UCO cohort. While intra-ventricular hemorrhage (IVH) is relatively uncommon in full-term neonates, it has been noted to occur with maternal abdominal compression, birth trauma and perinatal asphyxia [44]. Most parenchymal bleeding in the term infant involves the thalamus and is associated with IVH [45], and it has been suggested that thalamic hemorrhage in the late gestation fetus is the precursor to IVH at this developmental age [46]. The results of the current study show that brief global fetal asphyxia is sufficient to cause of intra-parenchymal haemorrhage in the late gestation fetus. It is possible that the small size of most of the microbleeds that we observed might not be visible on conventional ultrasound or MRI examination.

Several limitations of this study need to be acknowledged. Firstly, the behavioural observations made only up to 24 h after birth may not fully assess the consequence for the lamb of the asphyxial insult sustained at 132 days gestation. While there were no obvious signs of seizure in the UCO lambs, we cannot exclude that this did not occur; the EEG was not measured as this would have meant connecting the lamb to recording apparatus which would have interfered with lamb behaviour and lamb-ewe interactions. In other respects, the particular behaviours that we monitored, which reflect the ability of the lamb to resuscitate and feed itself, are as close as possible to those that would be made by neonatologists in their immediate assessment of the health of the newborn infant. Similar behavioural milestones have been used in previous studies to monitor lamb well-being [23] and determine “lamb vigour” shortly after birth [47]. Another consideration in our experimental design was that, given that some lambs show significant delay or failure to achieve these early behaviours, attempting to assess their behaviour over a period of several days may not be feasible without human intervention. In addition, a 24 h period was chosen for this study in order to assess the extent of any tissue damage present before any further changes could arise due to the problems with maternal care, mother-lamb bonding, or neonatal nutrition [22].

Secondly, we were not able to determine the true length of labour, which might have contributed to some of the variability observed between the UCO lambs. Delivery of the lambs by caesarean section would have removed this limitation, but the necessary use of an anaesthetic agent (general, epidural, or spinal) and the need to resuscitate the neonatal lamb would have prevented examination of the normal process of spontaneous recovery of the lamb at birth, and removed the opportunity to examine ewe-lamb interaction that leads ultimately to the lamb managing to find the teat and begin to suckle. Indeed, our main aim was to see to what extent the capacity for normal behaviour in lambs at birth had been restricted by the preceding event of brief *in utero* asphyxia.

Thirdly, the fetuses in the UCO group were subjected to frequent blood sampling before and after occlusion of the umbilical cord whereas the full-term and preterm control lambs were not. The 10 arterial blood samples (2–3 ml) taken over 12 h (see Methods for details of times), and further samples at 24 and 48 h after UCO, represent a blood loss of up to 7.3%, based on the birth weight of 3.52 kg for these fetuses, and assuming a blood volume of 140 ml/kg [48]. Despite this blood loss (the greatest relative loss would have been 3% for the 5 samples taken between −5 and +60 mins with respect to the UCO), the fetuses did not become hypotensive or show evidence of hypovolaemia, indicated by the maintenance of arterial pressure and haematocrit. In previous studies where control (sham) fetuses were subjected to the same blood sampling protocol as UCO fetuses, we noted significant changes in fetal behaviour, brain pathology and protein expression only in the fetuses subjected to UCO [10], [11], [15]. Nevertheless, it is possible that this blood taking procedure contributed to the some of the differences in lamb behaviour and brain pathology in the UCO group, compared to the full-term and preterm lambs, noted in the present study.

In pursuit of a relevant animal model of perinatal hypoxic-ischemic brain injury, the present sheep study can be added to those which use the pregnant macaque monkey [18] and rabbit [33], [49], where the consequences for the neonate of injury to the late gestation *fetal* brain have been studied. While our results support that a *in utero* asphyxic episode delivered some time (approx. 15 days) before the expected time of delivery was strongly associated with behavioral deficiencies and brain pathology in the neonate, it has not been possible to differentiate between: (i) the severity of injury caused at the time of insult; (ii) additional injury that may arise at birth as a legacy of the original insult; and (iii) cellular responses that are initiated to repair the damaged fetal brain. This may explain some of the variability we observed in the lambs brains at 24 h after birth. UCO in fetal sheep has been shown to increase expression of EPO [15], BDNF [17], and vascular endothelial growth factor [50], which may reflect processes that attempt to protect and repair the brain [23], [50]. However, the presence of established cystic lesions, regions of acellularity, and loss of oligodendrocytes with reduced myelination all suggests that these injuries occurred previously, at the time UCO was used to cause the fetal asphyxia. On the other hand, the presence of micro-bleeds and intra-parenchymal albumin is evidence of more recent damage to the brain, possibly arising at birth. These data therefore suggest that the insult itself directly causes a cascade of cellular damage, but also increases the vulnerability of the developing brain to further injury.

In conclusion, the present study provides evidence that a brief, ‘sentinel’ event incorporating global fetal hypoxia, hypercapnia and acidemia in late gestation results in brain damage and neurodevelopmental delays at birth, and may be associated with premature birth. Our data also shows that preterm birth accounts for some of the delay in achieving some of these milestones. The neuropathology observed in the brains of UCO lambs 24 hours after birth corresponds with some of the anatomical and pathological abnormalities reported in cerebral palsy, and is consistent with the ’typical’ morphological changes seen in acute brain inflammation and demyelination. These results inform us that brief *in utero* asphyxia in sheep has consequences that include preterm birth, neuro-functional deficits, and neuropathology that persist until after birth. These findings have important implications for the quest to find clinically useful and relevant treatments that protect the fetal and neonatal brain.
